# Anæsthetic Inhalation

**Published:** 1870-04

**Authors:** 


*Anœsthetic Inhalation.*—At a recent meeting of the Town Council of Edinburg, Scotland, the freedom of the City was presented to Sir, James Y. Simpson, Bart. M. D. on which occasion the Lord Provost in a speech made use of the following expression "I will not dwell on what you have accomplished in medical science, I will only allude to your discovery,—the greatest of all discoveries in modern times,—the applicatiou of chloroform to the assuagement of human suffering."

To this Prof. J. Bigelow of Boston takes exception and in an article to the *Boston Med. & Surgical Journal*, says: "Sir. James Simpson, in a long and eloquent reply to the Lord Provost, while he complacently accepts the crown of borrowed plumes thus tendered to him, makes not the slightest allusion to the country from which they were plucked, in which country anæsthetic inhalation, with more agents than one, were established, vindicated, and successfully practiced long before it was heard of in Edinburg or any part of Europe." "The history of anæsthetic inhalation is well known. It began in this country, and was first used in the extraction of teeth, and afterwards in capital operations in the Massachusetts General Hospital, and in obstetrical practice. The attention of the civilized world was immediately drawn to the great American discovery."

Dr. Simpson replies to the letter of Dr. Bigelow by one sent to Prof. Elliot, of New York, for publication in the *N. Y. Med. Journal*, in which he says: "I have year after year heartily paid every due compliment to the most important part borne in the consumation of the practical application of anæsthetics by America, particularly by the cities of Hartford and Boston, and especially by the energy and genius of Dr. Morton. Surely, it would have been sadly out of place on such an occasion and with such an audience to have shown that, before I discovered the application of chloroform to anæsthetic purposes, numerous other agents had been previously suggested and used for the same object,—as sulphuric ether by Drs. Jackson, Morton, and Marcy—as carbonic acid by Dr. Hickman, in imitation of the experiments performed for ages on the poor dogs at the Grotto del Cani, and as nitrous oxide (an agent extensively employed as a dentist's anæsthetic at the present hour,) and which was first proposed some seventy years ago for "destroying physical pains during surgical operations" by Sir Humphrey Davy.

"In the way of a climax, you terminate one of the paragraphs in your letter with the statement that I was not the "first man" to inhale a vapor to such an extent as to destroy sensibility. Most certainly I was not, and certainly I never was so intensely foolish as to claim to be so. In the course of my investigation I have, however, experimented upon myself with various vapors, the innocuous or the poisonous effects of which upon the economy were previously altogether unknown and unascertained, and I have sometimes suffered in consequence. As a professor of therapeutics, you must surely be well aware that the first experiment of breathing a vapor to such an extent as to destroy sensibility was made neither in America nor in our own days. Withont adverting to the acknowledged fact that it was accomplished with the vapors driven off from hypnotic vegetable extracts by other surgeons, from Hugo de Lucca and Theodoric downward, let me remind you that Sir Humphrey Davy boldly (and notwithstanding that he had witnessed occasional deaths on animals from it) made the experiment to which you advert many times upon himself in the last year of the last century with nitrous oxide, and found that headache and other pains disappeared under its influence.

About forty years ago Faraday, in this country, and Godman in America, showed, as the result of their observations and experience, that the effects of the inhalation of the vapor of sulphuric ether were quite similar on the nervous system to those produced by the inhalation of the vapor of nitrous oxide gas—a truth subsequently proved by many pupils in many chemical and other schools in your country, as well as in mine, by their inhalation of ether. Your remarks, so far as I understand them, imply that it is your belief that Dr. Morton was the "first man" of "sufficient courage" to breathe "a vapor" so as to produce a state of anæsthesia. But you must know as well as I do, from the official documents laid before the senate of the United States, that this is doubtful as regards the course of matters even in America. For it appears on these documents—1. That Dr. Jackson avers that he breathed for this object sulphuric ether earlier than Dr. Morton. 2. That before Dr. Morton made the same experiment upon himself, in 1846, he made it first upon others, and particularly upon his pupil, Mr. Speirs; and 3. That two years previously, or in 1844. Dr Marcy, of Hartford, had successfully excised a tumor from a man who had been rendered anæsthetic for the purpose, by the vapor of sulphuric ether, while at that same early date, in the same city, Dr. Horace Wells had extracted teeth from a dozen or more patients rendered insensible by inhaling nitrous oxide according to Davy's suggestion.

There has lately been raised, I am told, in the city of Boston, a monument, in commemoration of the employment of anæsthesia in surgery in that city in 1846. But have the erectors of this monument cut upon it the names of either of your fellow-citizens, Dr. Morton or Dr. Jackson, as the first investigators, or the names of Warren and Hayward, as the first Boston hospital surgeons who operated upon patients under the influence of sulphuric ether? Or have they generously inscribed upon its sides any allusion to the fact that two years previously anæsthetics had been inhaled successfully in dentistry and surgery in the neighboring city of Hartford? I have been assured—though it is scarcely credible—that there does not appear upon the monument the name of a single American chemist, dentist, or surgeon. Why is it so? You have the monument. Have you not had the men?

Dr. Bigelow replies to this letter of Dr. Simpson in the *Boston Medical & Surgical Journal*, and closes as follows:

" Finally you allude to the monument erected in Boston by a public spirited individual, and which, among others, bears the following inscription. " To commemorate the discovery that the inhaling of ether causes insensibility to pain; first proved to the world at the Massachusetts General Hospital, in Boston, October, A. D. 1846." You inquire why no individual names were inscribed upon it. I reply, because it was intended only to commemorate the city of Boston as the birth-place of the discovery.

Mankind are not apt to forget their benefactors. They cheerfully unite in ovations and festivities given to distinguished men by "their friends and fellow citizens." But the suffering and now exempted world, will not forget the poor dentist, who amid poverty, privation and discouragement, matured, revealed and established the most beneficent discovery which has blessed humanity since the primeval days of Paradise."

